# Pancreaticopleural fistula in children with chronic pancreatitis: a case report and literature review

**DOI:** 10.1186/s12887-020-02174-x

**Published:** 2020-06-03

**Authors:** Jia-yu Zhang, Zhao-hui Deng, Biao Gong

**Affiliations:** 1 Department of Pediatric Digestive Diseases, Shanghai Children’s Medical Center, Shanghai Jiao Tong University School of Medicine, Shanghai, 200127 China; 2 Department of Digestive Diseases, Shanghai Shuguang Hospital, Shanghai University of Chinese Medicine, Shanghai, 201203 China

**Keywords:** Pancreaticopleural fistula, Chronic pancreatitis, Child, Case report

## Abstract

**Background:**

Pancreaticopleural fistula (PPF) is a very rare and critical complication of pancreatitis in children. The majority of publications relevant to PPF are case reports. No pooled analyses of PPF cases are available. Little is known about the pathogenesis and optimal therapeutic schedule. The purpose of this study was to identify the pathogenesis and optimal therapeutic schedule of PPF in children.

**Case presentation:**

The patient was a 13-year-old girl who suffered from intermittent chest tightness and dyspnea for more than 3 months; she was found to have chronic pancreatitis complicated by PPF. The genetic screening revealed SPINK1 mutation. She was treated with endoscopic retrograde cholangiopancreatography (ERCP) and endoscopic retrograde pancreatic drainage (ERPD); her symptoms improved dramatically after the procedures.

**Conclusions:**

PPF is a rare pancreatic complication in children and causes significant pulmonary symptoms that can be misdiagnosed frequently. PPF in children is mainly associated with chronic pancreatitis (CP); therefore, we highlight the importance of genetic testing. Endoscopic treatment is recommended when conservative treatment is ineffective.

## Background

Pancreaticopleural fistula (PPF) is a very rare critical complication of pancreatitis in children that may occur secondary to acute or chronic pancreatitis, external or iatrogenic pancreatic trauma, leading to a fistula connecting the pancreas and pleural cavity presented or direct extension of a pseudocyst occurs when pancreatic duct rupture or pseudocyst formation; this can cause massive recurrent pleural effusion through the diaphragmatic hiatus and the peridiaphragmatic lymphatic plexus [1]. PPF causes significant pulmonary symptoms; it is misdiagnosed frequently, leading to a prolonged hospitalization time. In contrast to adult chronic pancreatitis (CP), wherein smoking and alcohol are important risk factors, genetic predisposition is a major cause of CP in children [2]. As significant differences were observed in the forward prognosis among the patients with and without mutations [3–7], it is important to definite the cause of PPF, and determine the risk factors of primary pancreatic disease for the long-term follow-up. At present, no pooled analyses of PPF cases are available. Little is known about the pathogenesis and optimal therapeutic schedule. Here we describe a case of PPF in a girl who suffered from chest tightness, dyspnea, and massive pleural effusion and was successfully treated through endoscopic procedures after failed conservative therapy. The objective of this report was to identify the pathogenesis and optimal therapeutic schedule of pancreaticopleural fistulas in children by reviewing relevant literature.

## Case presentation

A 13-year-old girl presented with intermittent chest tightness and dyspnea for 3 months. She was admitted to a local hospital twice. On her first admission, blood smear examination showed a significantly increased eosinophilic ratio, and the cysticercus antibody was weakly positive. Chest and abdomen computed tomography (CT) showed a little left pleural effusion, uneven density of pancreas, and pelvic effusion. She was treated with albendazole, but the girl failed to follow medical advice, she stopped taking medicine after 5 days. Ten days later, her chest tightness and dyspnea aggravated, so she was readmitted to the hospital, chest CT showed a large left pleural effusion with atelectasis. She was then treated with thoracic tube drainage and albendazole. After 2 weeks, her chest tightness and dyspnea improved. However, she still complained of intermittent chest tightness and dyspnea within 2 months after discharge and lost 5 kg in the last six months. To further clarify the cause, the girl was referred to our hospital. In fact, she was complaining of intermittent abdominal pain for more than 1 year; however, since the pain was not intense, her parents did not pay attention to the complaint. The patient did not have any bad habits, such as smoking or drinking, and she had no history of abdominal trauma and surgery and biliary and pancreatic diseases. Her parents, sister, and brother were all in good health.

**Fig. 1:**
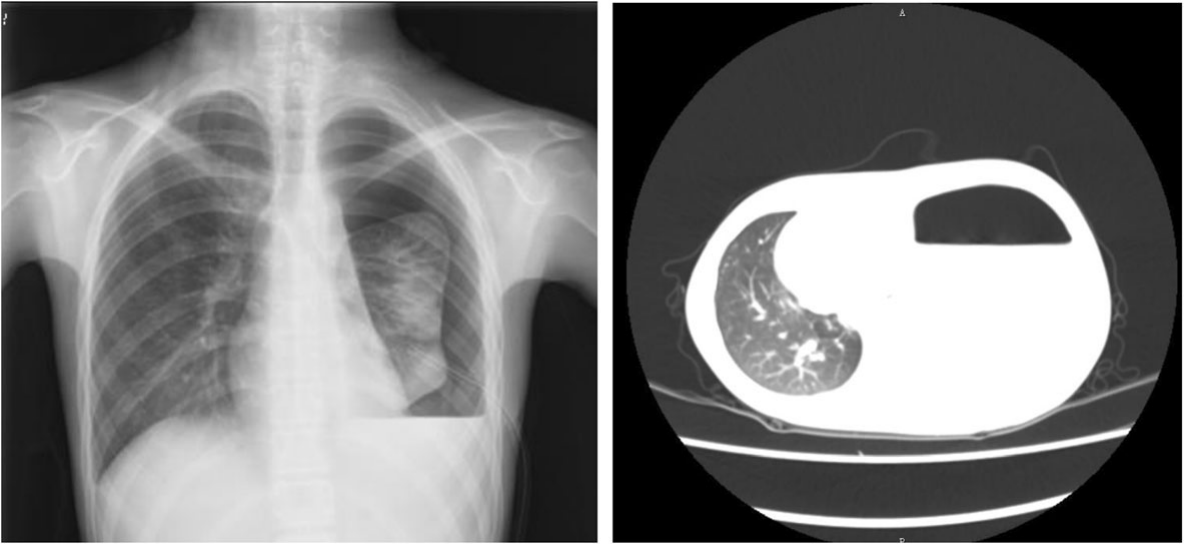
An air-fluid level and atelectasis can be seen on the chest x-ray (left) and computed tomography (right) images, which showing massive left hydropneumothorax

**Fig. 2:**
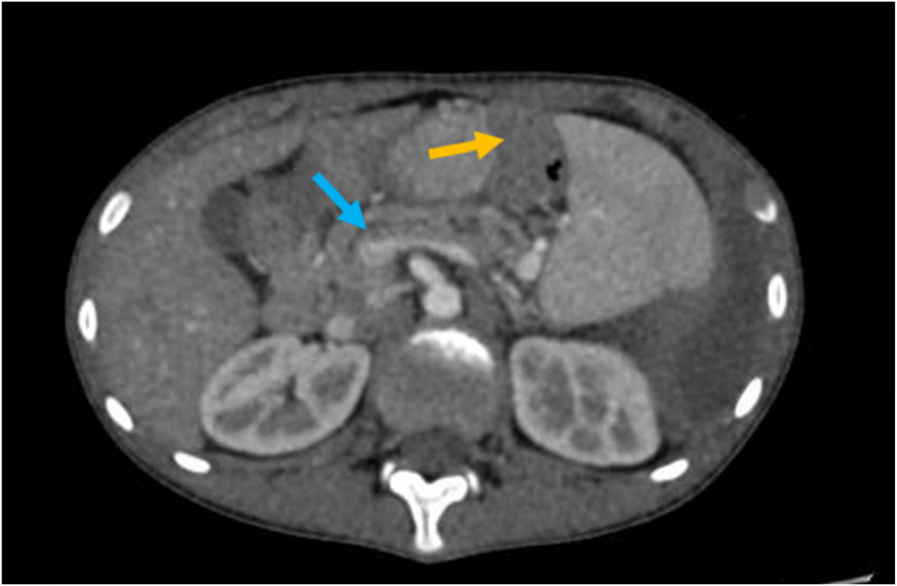
Abdominal CT showed a small low-density lesion at the distal pancreas, accompanied by a dilatation of the main pancreatic duct (blue arrow) and the pancreatic pseudocyst (yellow arrow)

**Fig. 3:**
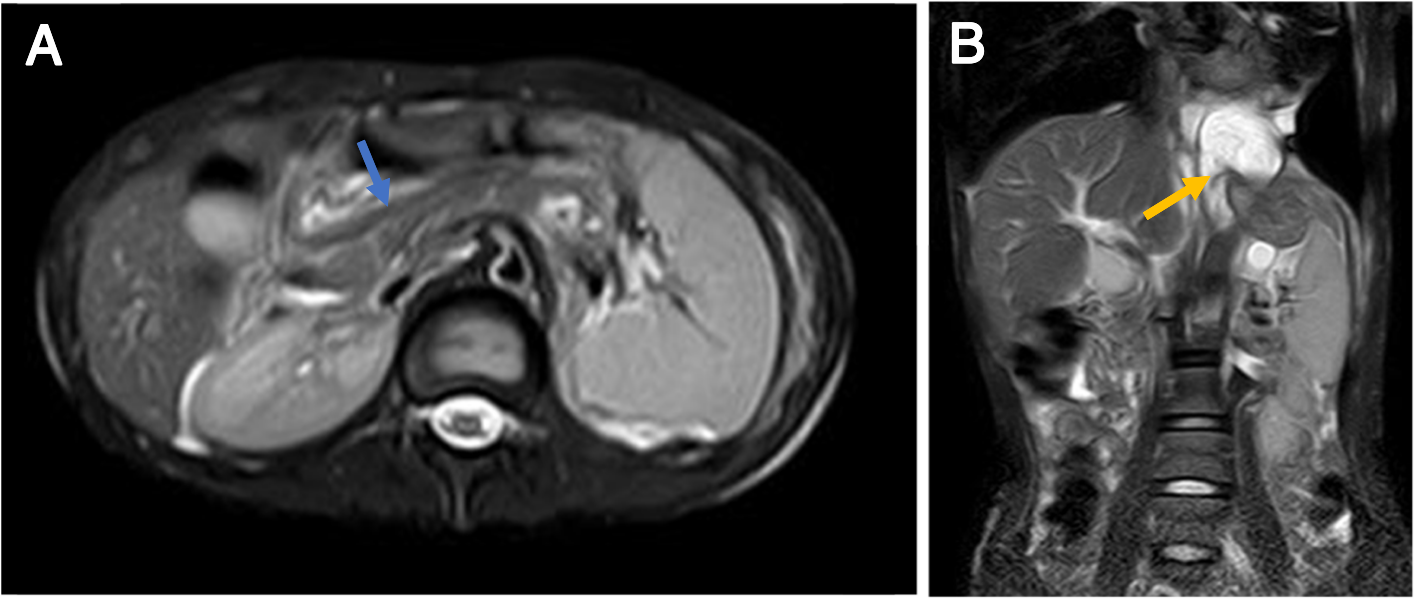
**a** An MRCP revealed dilatation of the main pancreatic duct (blue arrow). **b** An MRCP revealed an abnormal tubular structure from the pancreatic pseudocyst to the pleural cavity (yellow arrow)

The patient’s height and weight were 165 cm and 36 kg, respectively. Physical examination revealed decreased vocal fremitus and breath sounds and dullness to percussion on the left hemithorax. Other components of her physical examination were unremarkable. Serum revealed mildly elevated amylase levels of 193 IU/L and lipase levels of 536 IU/L, whereas pleural fluid amylase was elevated with levels of > 2400 IU/L. Chest x-ray and thoracic CT scan confirmed massive left hydropneumothorax with atelectasis (Fig. 1). Abdominal CT scan showed a small low-density lesion at the distal pancreas, accompanied by a pancreatic pseudocyst and main pancreatic duct dilatation (Fig. 2). Subsequently, magnetic resonance cholangiopancreatography (MRCP) revealed an abnormal tubular structure extending from the pancreatic pseudocyst along the spine to the pleural cavity, which was considered as a fistulous tract (Fig. 3). Hence, due to the radiological appearance and elevated pleural fluid amylase, massive recurrent pleural effusion was thought to be secondary to PPF, which was a complication of chronic pancreatitis. The patient and her parents underwent genetic tests, which revealed that the SPINK1 gene had “splice site variation c.194+2T> c (heterozygosity)”. The mother carried this site variation (heterozygosity), while her father had a normal genotype.

**Fig. 4:**
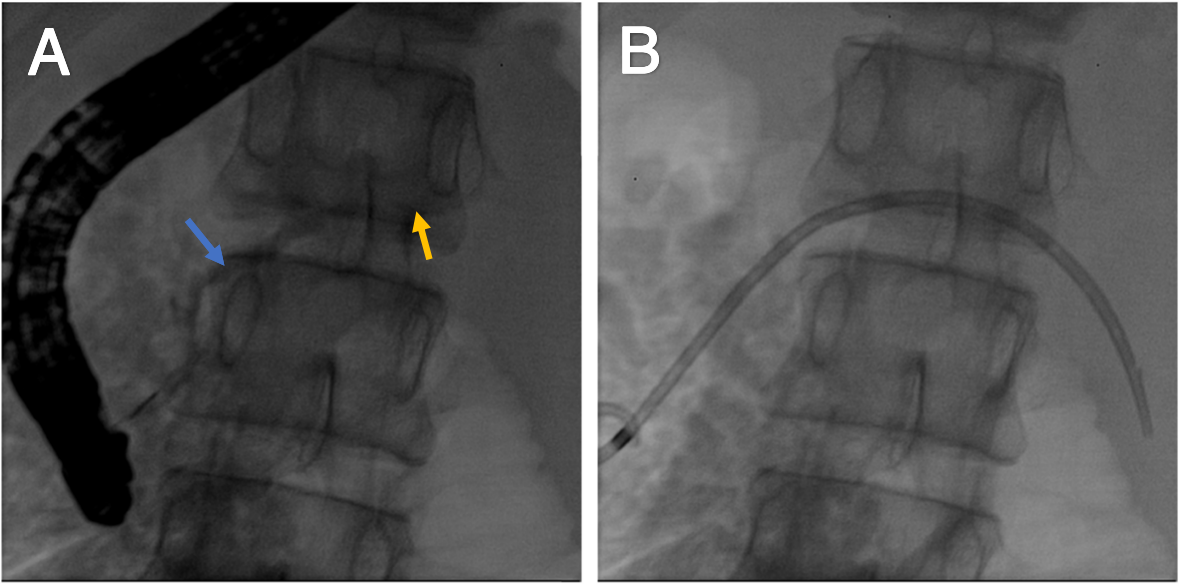
**a** Endoscopic retrograde cholangiopancreatography (ERCP) showed segmental stenosis and dilatation of the pancreatic duct (blue arrow) and a pseudocyst at the pancreatic body and tail (yellow arrow). **b** ERCP showed a stent was placed into the pancreatic duct

A pleural drain was maintained for the patient. For fasting conditions, total parenteral nutrition was followed, and somatostatin and ulinastatin were initiated for 12 days. However, she still complained of intermittent chest tightness; bloody fluid continued to flow out from the chest drainage tube. The patient then underwent an endoscopic retrograde cholangiopancreatography (ERCP) that showed segmental stenosis and dilatation of the pancreatic duct and a pseudocyst at the pancreatic body and tail (Fig. 4). Endoscopic retrograde pancreatic drainage was performed. Two days later, there was a relief of chest tightness, and pleural effusion was significantly reduced. Due to the intractable pneumothorax, erythromycin was injected into the pleural cavity to fix the pleura for 5 days. Thirty- seven days after ERCP, the pleural drain was removed, and the patient was discharged at hospital day 52. Chest x-ray and serum amylase of the patient was followed-up regularly for 5 months, eventually revealing normal results. Five months after discharge, abdominal CT showed that the pancreatic pseudocyst was completely cured. Another ERCP was performed, which showed segmental stenosis and dilatation of the pancreatic duct, and the pseudocyst disappeared; hence, nasopancreatic drainage was performed for 3 days after the pancreatic duct stent was removed.

### Study identification and statistical analysis

An extensive review of the literature was performed using the databases of PubMed, OVID, EMBASE, Medline, CNKI, and WANFANG, with keywords such as “pancreaticopleural fistula” and “child.” We retrospectively analyzed 22 cases, including the current case and 21 additional patients derived from six Chinese articles and eight English articles (Table 1).

**Table 1:** Literature review of children with pancreaticopleural fistula

Study	N; age (years)/ Gender	Etiology	Genetic test	Main complaint	Pleural fluid amylase^#^	Serum amylase^#^
Ozbek et al. [8]	1;5/F	Trauma	None	Abdominal pain, dyspnea	1200	334
G Tanir et al. [9]	1;12/M	Trauma	None	Thoracalgia, abdominal pain, dyspnea	–	318
Lee et al. [10]	1; 3.2 /M	CP	PRSS1 gene mutation	Abdominal pain, dyspnea	25,460	888
Duncan et al. [11]	2;1.6/M,10/M	CP	None	Dyspnea (2 cases), abdominal pain (1 case)	950, 157,000	Normal, Not clear
Bishop et al. [12]	1;4/F	CP	Negative	Dyspnea, wheeze	12,170	751
Ranuh et al. [13]	1;12/M	CP	None	Abdominal pain, dyspnea	40,000^&^	1974^&^
Fitzgibbons et al. [14]	1;16/F	CP	None	Thoracalgia, abdominal pain, dyspnea	45,666	–
Wakefield et al. [15]	2;3/M,4/M	?Congenital ductal anomaly	None	Abdominal pain in 2 cases, dyspnea in 1 case	9737, > 16,000	329,4935
Zhuang LL et al. [16]	1;14/F	CP	None	Cough, chest pain, dyspnea	11,239.8	566.6
Liu XY et al. [17]	1;14/M	CP	None	Cough, dyspnea	26,110	1911
Yu FH et al. [18]	5;2 ~ 10.4/M*3,F*2	CP	None	Chest tightness, chest pain, fever in 3 cases, wheezing, dyspnea, abdominal pain in 1 case	1546 ~ 50,465	110 ~ 889
Li J et al. [19]	1;11/F	CP	None	Chest tightness	4206	130
Chen B et al. [20]	2;2/M,8/M	Trauma	None	Fever in 2 cases, abdominal distension, cough, dyspnea in 1 case	> 1300	Not clear, 5100
Yu ZX et al. [21]	1;8/F	CP	None	Dyspnea	56,365.7	504.8

Note: #: IU/L; &: lipase

All available data were entered into a customized database and then analyzed by SPSS software version 23.0 (IBM Corp, Armonk, NY, USA), quantitative data were summarized as mean ± standard deviation (SD) or number with percentage, where appropriate. Statistical analysis was performed using independent t-test, one-way ANOVA test, and Tukey’s post hoc test; statistical significance was defined as *P* < 0.05.

The mean time to diagnose PPF was 2.69 (0.25 ~ 6) months. Etiology analysis revealed 17 cases (77.3%) of CP, 4 cases (18.2%) of traumatic pancreatitis and one case (4.5%) of suspected congenital ductal anomaly. In addition, 16 of 22 cases accompanied by a pancreatic pseudocyst. Among the 22 cases, 3 cases had complete genetic tests; one case revealed SPINK1 gene mutation, and one case revealed PRSS1 gene mutation. The main manifestations were dyspnea (15 cases, 68.2%), abdominal pain (8 cases, 36.4%), and thoracalgia (6 cases, 27.3%). Except for three patients who were not clearly reported, amylase levels of the pleural effusion were significantly increased (950 ~ 157,000 U/L) in other patients. Seventeen cases (77.3%) of fistula can be diagnosed by complementary imaging tests; among the 17 patients, only 9 cases (53%) of fistula and its anatomy were identified through the esophageal hiatus (6 cases) and the aortic hiatus (3 cases) extending to the thoracic cavity. CT scan was performed in 14 cases, but fistulas were only found in 8 cases, with a sensitivity of 57.1%; MRCP was performed in 9 cases, then 7 cases showed fistula, with a sensitivity of 77.8%; ERCP was performed in 12 cases, of which 7 cases were therapeutic operations, and 5 cases were diagnostic operations, only 3 cases showed fistula, with a sensitivity of 25%. Three cases (13.6%) of fistula were confirmed during surgery; 2 cases (9.1%) of fistula could not be demonstrated by imaging tests or surgical operation. Surgery alone was performed in four cases. Eighteen cases were first managed with conservative treatment; however, 14 cases needed endoscopic treatment (7 cases) or surgical intervention (7 cases) (Table 2).

**Table 2:** Baseline characteristics of children with pancreaticopleural fistula (*n* = 22)

	No	%
Demographics		
Male	13	59.1
Etiology		
CP	17	77.3
traumatic	4	18.2
?Congenital ductal anomaly	1	4.5
Accompanied by pancreatic pseudocyst	16	72.7
Main manifestations		
dyspnea	15	68.2
abdominal pain	8	36.4
thoracalgia	6	27.3
Diagnosis of fistula		
Imaging tests	17	77.3
Surgery	3	13.6
No fistula could be demonstrated	2	9.1
Conservative treatment	4	18.2
Endoscopic treatment		
ERPD	3	13.6
EST + EPBD	1	4.5
ERPD+ Stone extraction	1	4.5
EST+ Stone extraction+ ERPD	1	4.5
Nasopancreatic drainage followed by stenting of the duct	1	4.5
Surgery treatment		
LPJ	8	36.4
Internal drainage of pseudocysts and anastomosed to a Roux-en-Y loop of jejunum	1	4.5
Partial pancreatectomy	1	4.5
Partial pancreatectomy, pancreatolithotomy and LPJ	1	4.5

Note: *EST* Endoscopic sphincterotomy; *ERPD* Endoscopic Retrograde Pancreatic Drainage; *EPBD* Endoscopic Papilia-sphincter Balloon Dilatation; *LPJ* Longitudinal pancreaticojejunostomy

Endoscopic treatment is a safe therapeutic option, among the 7 cases, only one case needed to reset a stent due to the pancreatic stent was removed spontaneously via defecation 8 days after stent insertion. However, one patient had empyema and bleeding after surgery. The efficacy of endoscopic treatment has also been proven; through endoscopic treatment, clinical symptoms and pleural effusion were improved significantly after 4 ± 1.6 days, compared with 5 ± 2.8 days after surgical intervention, there were no statistical differences; but compared with 17 ± 4 days after conservative treatment, statistical differences could be seen(*p* = 0.02). All patients improved and were discharged; the mean hospitalization time of endoscopic treatment was 34 ± 17 days, and conservative treatment was 50 ± 12 days, there were no statistical differences between the two groups. It’s because endoscopic treatment was carried out after ineffective conservative treatment; the hospitalization time would have been prolonged. Patients treated by endoscopic treatment were in good health within three to fourteen-months follow-up, and those treated by surgical intervention also remained healthy within eleven to twenty-four months follow-up. Unfortunately, the hospitalization time of surgical intervention and follow-up information about conservative treatment could not acquire from our review, so that no more analysis can be made.

## Discussion and conclusions

PPF is a rare complication of pancreatitis. It is caused by acute or chronic pancreatitis, pancreatic trauma, or iatrogenic rupture of the pancreatic duct. Among the 22 cases of PPF, 17 cases (77.3%) were secondary to chronic pancreatitis, indicating that chronic pancreatitis was the main cause of PPF in children. Adult CP is mainly due to acquired factors, such as alcohol and smoking. CP in children is mostly associated with gene mutation and abnormal structure of the biliopancreatic duct. Gene mutation is the main risk factor of CP in children. Previous research in children has shown that 33% with acute pancreatitis (AP), 45.4% of acute recurrent pancreatitis (ARP), and 54.4% with CP have genetic susceptibility [22]. Xiao Y et al. [23] found that the positive rates of pathogenic genes for CP and ARP in Chinese children were 71.1 and 47.1%, respectively. In our review, three children with CP underwent genetic testing, and two of them revealed gene mutations. This indicates that children with CP may have genetic abnormalities that are closely related to the development of CP. Hereditary pancreatitis is a dominant inheritance with high penetrance, which may be complicated with pancreatic exocrine dysfunction (35–37%), diabetes (26–32%), and pancreatic cancer (6%) in the future [3, 4]. Mutation-positive patients had significantly earlier median ages at diagnosis of pancreatic stones, diabetes mellitus, and steatorrhea than mutation-negative CP patients [5]. In addition, children with mutation-positive reveal a significantly more severe clinical course of the disease and complications than mutation-negative children [6, 7]. Therefore, genetic testing has important significance for predicting prognosis and long-term management in children.

Currently identified pathogenic genes include serine protease inhibitor Kazal type 1 gene (SPINKl), cystic fibrosis transmembrane conductance regulator gene (CFTR), cationic trypsinogen protease serine 1 (PRSS1) gene, and the cystic fibrosis transmembrane conductance regulator gene (CTRC) gene [24]. The genetic basis of CP varies significantly according to age, race, and region [25, 26]. The mutation rate of the PRSS1 gene in Chinese children with chronic pancreatitis is significantly higher than in adults. The IVS3 + 2TC splice site mutation of SPINK1 is the most common gene mutation in Chinese children [18], while the N34S gene mutation of SPINK1 is most common in white patients [27–30]. In the present study, two patients revealed gene mutations; one case was reported in Korea, revealing an R122H mutation of PRSS1 gene with a family history of pancreatic disease, and the other case is our patient with “splicing site variation c.194+2T> c (heterozygous)” mutation of SPINK1 gene.

Diagnosing PPF is not complex; it can be diagnosed through significantly elevated amylase in the pleural effusion and through abdominal imaging test. However, it can still be misdiagnosed frequently. The average time to diagnosis PPF is 5 weeks based on the previous study [31]. The main reason for misdiagnosing is that PPF is a rare disease, and the main manifestations are pulmonary symptoms caused by repeated pleural effusion, and abdominal symptoms are infrequent. Sometimes, serum amylase may not be increased, and the fistula can be difficult to demonstrate radiologically. In this study, 77.3% of fistulas can be demonstrated radiologically; MRCP is the best imaging test to diagnose PPF with a sensitivity of 77.8%, which is consistent with previous research [32], and no radiation. The anatomical relationship between the pancreatic duct and the fistula can also be demonstrated in detail, which is beneficial to determine therapy; CT scan can better reveal the pancreatic parenchyma with a sensitivity of 57.1%. However, the sensitivity of ERCP to demonstrated PPF is 25%, which is significantly lower than the previous study [33]. ERCP is superior to other modalities to show the pancreatic anatomy but will often fail to demonstrate the fistula, selective duct cannulation, or even an operative pancreatogram may be required in the presence of tight structure [34]. In our study, only 53% of PPF and its anatomy were identified through imaging, which showed that imaging test is limited in revealing the anatomy of PPF. The main approaches of PPF to the mediastinum are aortic hiatus and esophageal hiatus. Imaging tests can show the diffusion pathway of the retroperitoneal space; however, it cannot show the relationship between the fascia plane, ligament, and retroperitoneal subspace clearly, which is the reason for the limitation of imaging test.

The treatment of PPF includes conservative treatment, endoscopic treatment, and surgical intervention. The treatment depends on the ductal anatomy. A normal or mildly dilated pancreatic duct, including traumatic pancreatitis, can be managed with conservative treatment, including pleural drain, trypsin inhibitor, nasojejunal tube feeding, and total parenteral nutrition. In 30–60% of cases, medical treatment is successful [35, 36]. In the presence of ductal incomplete disruption in the head or body of pancreas and distal stricture, an endoscopic approach can be made initially using a stent, sphincterotomy, or balloon dilatation, which can reduce the pressure of the pancreatic duct. In 88% of cases, pancreatic duct fractures can heal [37], and 48% of fistulas can be closed within 2–3 weeks [38, 39]. If endoscopic treatment is not possible due to complete ductal disruption, ductal obstruction proximal to fistula, leak in the tail region, or unsuccessful management, surgery, such as partial pancreatectomy, longitudinal pancreaticojejunostomy (LPJ), or internal drainage of pseudocysts can be considered [33]. PPF is a rare complication in children; there are no relevant epidemiological studies to confirm which therapeutic method is the best. In the present study, 18 cases were treated with conservative treatment initially; however, only one case of CP and 3 cases of trauma pancreatitis with PPF could be managed successfully, the other 14 cases need endoscopic treatment and surgery intervention eventually, indicating that except for traumatic pancreatitis with PPF, the most PPF cannot be managed successfully with conservative treatment.

**Fig. 5:**
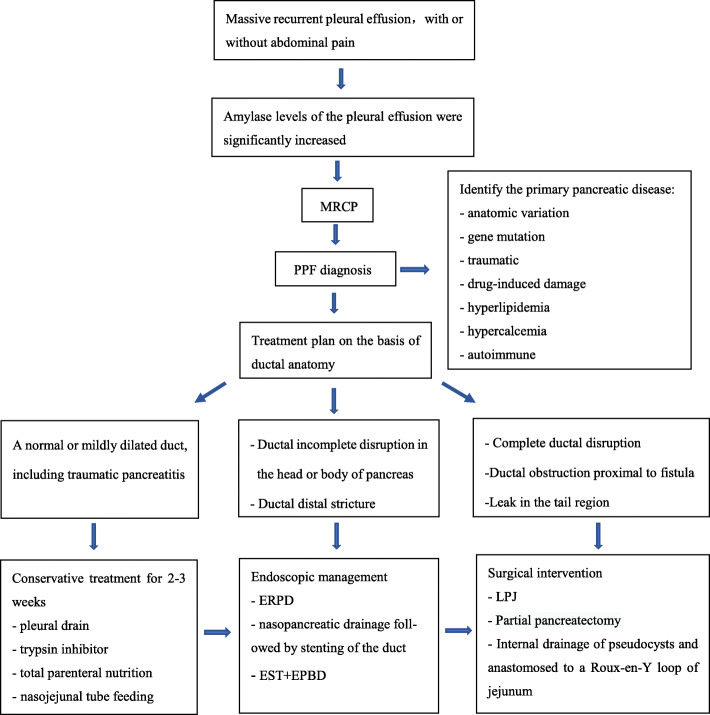
Flowchart for the treatment strategy in children with pancreaticopleural fistula

Surgical treatment for PPF mainly includes pancreatectomy and LPJ, but for the primary pancreatic disease, such as CP, there is a high rate of pain recurrence after operation [40], sometimes even cause pancreatic insufficiency. Compared with surgery, endoscopic treatment has the advantages of being minimally invasive, quick recovery, fast transition to enteral nutrition, which can be repeated and significantly shortened hospitalized time [41, 42]. Recently reported literature showed that endoscopic treatment for symptomatic CP in children is a safe and effective therapeutic option [43–45]. D Kohoutova et al. [46] recommend endoscopic treatment of CP in children before surgical operation based on their long-term follow-up. In this study, two cases of PPF with gene mutations were cured by endoscopic treatment. We found that endoscopic treatment was minimally invasive and effective. After placing a stent, pleural effusion was significantly reduced on the second day without any related complications, and the pancreatic tissue has no additional damages. During the five-months follow-up, she was in good health, symptom-free, and serum amylase level are within normal limits. Therefore, endoscopic treatment is recommended for PPF in children, especially for chronic pancreatitis. A flowchart for the optimal treatment strategy in children with PPF has been recommended (Fig. 5).

PPF is a rare pancreatic complication in children, which can be misdiagnosed frequently. It should be considered when a child presents with repeated massive pleural effusion. The etiology of PPF in children is mostly due to CP. Genetic testing should be carried out to identify gene mutations. Endoscopic treatment is minimally invasive, safe, and effective; therefore, it is recommended for children with PPF.

## Data Availability

The data presented in this article are available in the reference listed below.

## Abbreviations

PPF: Pancreaticopleural fistula
ERCP: Endoscopic retrograde cholangiopancreatography
CP: Chronic pancreatitis
CT: Computed tomography
MRCP: Magnetic resonance cholangiopancre-atography
EST: Endoscopic sphincterotomy
ERPD: Endoscopic retrograde pancreatic drainage
EPBD: Endoscopic papilia-sphincter balloon dilatation
LPJ: Longitudinal pancreaticojejunostomy
